# Equity and efficiency of medical and health service system in China

**DOI:** 10.1186/s12913-023-09025-2

**Published:** 2023-01-14

**Authors:** Na Zhao, Kai Chen

**Affiliations:** 1 Party School of Liaoning Provincial Committee of C.P.C, Shenyang, Liaoning, 110004 China; 2 School of Business Administration, Northeastern University, Shenyang, Liaoning, 110819 China

**Keywords:** Medical and health services system, China, Equity, Efficiency, Coordination index

## Abstract

**Background:**

Equity and efficiency are basic value dimensions to evaluate the effectiveness of China’s medical and health service system (MHS) reform and development. Coordinated development of equity and efficiency is necessary to realize high-quality development of medical and health services. This study aims to evaluate the equity, efficiency, and combined efforts in coordinating the MHS during 1991–2020 reform.

**Methods:**

Data on China’s MHS were obtained from the *China Statistical Yearbook* 1992–2021. Ratios of urban to rural residents’ medical expenditure and number of medical professionals per 10,000 people were employed to evaluate MHS’s equity. The data envelopment analysis-Malmquist model was employed to evaluate MHS’s efficiency. We constructed a combined-efforts-in-coordination model to examine the coordination degree between equity and efficiency.

**Results:**

Equity of medical expenditure burden significantly improved from during 1991–2007. Urban residents’ 1991 medical expenditure burden was 87.8% of that of rural residents, which increased to 100.1% in 2007. Urban areas’ mean medical expenditure burden was 105.94% of that in rural areas during 1991–2007. The gap in equity of medical expenditure burden between urban and rural areas slowly widened after 2007, with urban areas’ mean burden being 68.52% of that in rural areas during 2007–2020. Medical and health resources allocation shows an alarming inequity during this period, with mean number of medical professionals per 10,000 people in urban areas being 238.30% of that in rural areas. Efficiency experienced several fluctuations before 2008. Since 2008, efficiency was high (0.915) and remained stable, except in 2020. The combined-efforts-in-coordination score for medical expenditure burden was less than 0.2 for 80% of the years, while that for in medical and health resources was more than 0.5 for 99.67% of the years.

**Conclusions:**

MHS inequity remains between urban and rural China, primarily because of disproportionate allocation of medical and health resources. The government should enhance rural medical professionals’ salary and welfare and provide medical subsidies for rural residents to adjust resource allocation levels in urban and rural areas, control differences in medical expenditure burden between urban and rural residents to a reasonable range, and continuously improve urban and rural residents’ equity level.

## Background

The Chinese government attaches significant importance to the development of an effective public health system [1]. A differentiated Medical and Health Service System (MHS) was established in urban and rural areas covering various social groups in both areas [2, 3]. This was an important measure to meet the medical demands of the general population based on productivity levels of that period. Chinese people’s demands for medical and health services changed as living conditions changed, which led to the reforming of China’s MHS [4, 5]. There has been a remarkable improvement in China’s MHS over the last decades. For example, the hierarchical diagnosis and treatment system has been established, and the universal medical insurance system has been improved [6, 7]. These changes have brought many benefits, including levels of improvement in medical services and expenditure burden reductions in medical treatments [8, 9]. As of 2020, China’s MHS encompassed 35,394 hospitals, 970,036 primary medical and health institutions, and 14,492 professional public health institutions [10]. As part of the achievements of the MHS reform, the coverage rate of secondary hospitals increased to 84% [11]. Based on the World Health Organization’s three recognized indicators measuring health levels in various countries, the life expectancy of Chinese people increased from 35 years in 1949 to 77.3 years in 2020, an increase of more than 40 years; China’s infant mortality rate dropped from 200‰ to 5.4‰; and the maternal mortality rate declined from 150/100,000 to 16.9/100,000 [12].

Although remarkable progress has been made in China’s MHS from 1985 to the present, challenges still remain. For example, there exists a wide gap between urban and rural medical and health resources [13–17], as well as critical problems such as low efficiency level of the overall medical and health services [18–21]. The imbalance and inefficiency of medical and health services not only violate the principle of maintaining social equity and justice, but also deviate from the target of common prosperity in China.

From the “Old Medical Reform” in 1994 to the “New Medical Reform” in 2009 and the “Comprehensive Reform of Public Hospitals” in 2017, the Chinese government remained committed to transforming the country’s public MHS. While striving to improve the MHS’s operational efficiency, China has continued to seek to reduce inequity in providing basic medical and health services for both urban and rural residents [22, 23].

Evaluation of the equity and efficiency of China’s MHS is important for health planners and decision-makers to identify bottlenecks and take appropriate actions to further optimize China’s MHS. This study aimed to assess the equity and efficiency of China’s MHS from 1991 to 2020 to explore potential measures for promoting equity and efficiency coordination development.

## Methods

### Data sources and statistical analysis

The data were sourced from the *China Statistical Yearbook* for 1992–2021. Since 1988, China has released annual data on hospital outpatient and inpatient expenses. Considering data availability and continuity, this study selected 1990 as the base year and defined the investigation period as 1991–2020. The scope of the analysis was limited to hospitals.

Microsoft Excel 2021 was used to calculate the equity, and DEAP2.1 was employed to conduct the data envelopment analysis (DEA)-Malmquist model.

### Equity of China’s MHS

The equity of medical insurance in China is measured by health expenditure burden and health resource allocation in urban and rural areas. Equity in health expenditure burden means that the ratio of urban residents’ medical and health expenditure to their disposable income is close to the ratio of rural residents’ medical and health expenditure to their disposable income. Equity in health resource allocation means that there is no substantial difference in the per-resident distribution of health resources among urban and rural residents. The formula to calculate the equity of China’s MHS is expressed as follows:

1 $${x}_{F}^{i}= \frac{{x}_{1}^{i}}{{x}_{2}^{i}}$$

$x_{F}^{i}$ is the equity ($i = 1$ denotes health expenditure burden; $i = 2$ denotes health resources allocation); ${x}_{1}^{i}$is the urban residents’ health expenditure burden or health resources; ${x}_{2}^{i}$ is the rural residents’ health expenditure burden or health resources. Equity in China’s MHS means that the medical expenditure burden and health resources allocation have gradually become consistent among urban and rural residents. That is, the closer the value of ${x}_{f}^{i}$ is to 1, the more equitable is China’s MHS.

### Efficiency of China’s MHS

To study the efficiency of China’s MHS, we applied the DEA method. DEA is a nonparametric performance evaluation technique commonly used to evaluate the relative efficiency of decision-making units with multiple input and output data. DEA uses linear programming, considers optimal input and output as the production frontier, and constructs the envelope curve. DEA can fully consider the optimal input–output solution of the decision-making units, display the information and features of the evaluated object, and play a unique role in analyzing the input–output of complex systems.

China’s MHS is a massive structure with multiple inputs and outputs, as well as complex correspondence between inputs and outputs. Considering that China’s MHS reform is complemented by continual coverage expansion, this study used the variable returns-to-scale Banker-Charnes-Cooper (BCC) model to evaluate its efficiency. The basic linear programming model of the BCC is given below:

2 $$min\left[\theta -\varepsilon \left({\sum }_{j=1}^{m}{S}^{-}+{\sum }_{j=1}^{r}{S}^{+}\right)\right], \left\{\begin{array}{c}{\sum }_{j=1}^{n}{\lambda }_{j}{x}_{j}+ {S}^{-}={\theta }_{x0}\\ {\sum }_{j=1}^{n}{\lambda }_{j}{y}_{j}+ {S}^{-}={\theta }_{y0}\\ \begin{array}{c}{\sum }_{j=1}^{n}{\lambda }_{j}=1\\ {\lambda }_{j},{S}^{-},{S}^{+} \ge 0, j=\mathrm{1,2},\dots , n\end{array}\end{array}\right.$$

The DEA-BCC model focuses on cross-sectional data. It can only compare efficiency levels that are horizontal and static at the same time node. It cannot comprehensively analyze panel data or measure dynamic changes and future development trends. If we had to only use the DEA-BCC model to analyze and evaluate the efficiency of China’s MHS, it would be difficult to comprehensively analyze the various efficiency changes over time. However, this study was conducted over an extended period, and it was imperative to account for temporal changes. We therefore introduced the Malmquist productivity index (MPI) method to analyze the panel data and demonstrate the dynamic changes in China’s MHS efficiency. The MPI is calculated based on the distance function (E) and is expressed using the following mathematical equations:

3 $$MPI_{I}^{t} = \frac{{E_{I}^{t} (x^{t + 1} ,y^{t + 1} )}}{{E_{I}^{t} (x^{t} ,y^{t} )}},MPI_{I}^{t + 1} = \frac{{E_{I}^{t + 1} (x^{t + 1} ,y^{t + 1} )}}{{E_{I}^{t + 1} (x^{t} ,y^{t} )}}$$

To fully comprehend the technical level of the two periods, we considered the geometric mean:

4 $$MPI_{I}^{G} = (MPI_{I}^{t} MPI_{I}^{t + 1} )^{\frac{1}{2}} = [\frac{{E_{I}^{t} (x^{t + 1} ,y^{t + 1} )}}{{E_{I}^{t} (x^{t} ,y^{t} )}} \times \frac{{E_{I}^{t + 1} (x^{t + 1} ,y^{t + 1} )}}{{E_{I}^{t + 1} (x^{t} ,y^{t} )}}]^{\frac{1}{2}}$$

The productivity function can be divided into input-oriented efficiency change (EFFCH) and technical change (TECHCH). Efficiency change can be subdivided into scale efficiency change (SECH) and pure efficiency change (PECH).

5 $$\begin{gathered} MPI_{I}^{G} = (EFFCH_{I} ) \times (TECHCH_{I}^{G} ) \hfill \\ = \frac{{E_{I}^{t} (x^{t + 1} ,y^{t + 1} )}}{{E_{I}^{t} (x^{t} ,y^{t} )}} \times [\frac{{E_{I}^{t} (x^{t + 1} ,y^{t + 1} )}}{{E_{I}^{t + 1} (x^{t} ,y^{t} )}} \times \frac{{E_{I}^{t} (x^{t + 1} ,y^{t + 1} )}}{{E_{I}^{t + 1} (x^{t} ,y^{t} )}}]^{\frac{1}{2}} \hfill \\ \end{gathered}$$

6 $$SECH = [\frac{{{{E_{vrs}^{t + 1} (x^{t + 1} ,y^{t + 1} )} \mathord{\left/ {\vphantom {{E_{vrs}^{t + 1} (x^{t + 1} ,y^{t + 1} )} {E_{crs}^{t + 1} (x^{t + 1} ,y^{t + 1} )}}} \right. \kern-0pt} {E_{crs}^{t + 1} (x^{t + 1} ,y^{t + 1} )}}}}{{{{E_{vrs}^{t + 1} (x^{t} ,y^{t} )} \mathord{\left/ {\vphantom {{E_{vrs}^{t + 1} (x^{t} ,y^{t} )} {E_{crs}^{t + 1} (x^{t} ,y^{t} )}}} \right. \kern-0pt} {E_{crs}^{t + 1} (x^{t} ,y^{t} )}}}} \times \frac{{{{E_{vrs}^{t} (x^{t + 1} ,y^{t + 1} )} \mathord{\left/ {\vphantom {{E_{vrs}^{t} (x^{t + 1} ,y^{t + 1} )} {E_{crs}^{t} (x^{t + 1} ,y^{t + 1} )}}} \right. \kern-0pt} {E_{crs}^{t} (x^{t + 1} ,y^{t + 1} )}}}}{{{{E_{vrs}^{t} (x^{t} ,y^{t} )} \mathord{\left/ {\vphantom {{E_{vrs}^{t} (x^{t} ,y^{t} )} {E_{crs}^{t} (x^{t} ,y^{t} )}}} \right. \kern-0pt} {E_{crs}^{t} (x^{t} ,y^{t} )}}}}]^{\frac{1}{2}}$$

7 $$PECH = \frac{{E_{vrs}^{t + 1} (x^{t + 1} ,y^{t + 1} )}}{{E_{crs}^{t + 1} (x^{t} ,y^{t} )}}$$

### Combined Efforts in the Coordination Model of China’s MHS

**Fig. 1:**
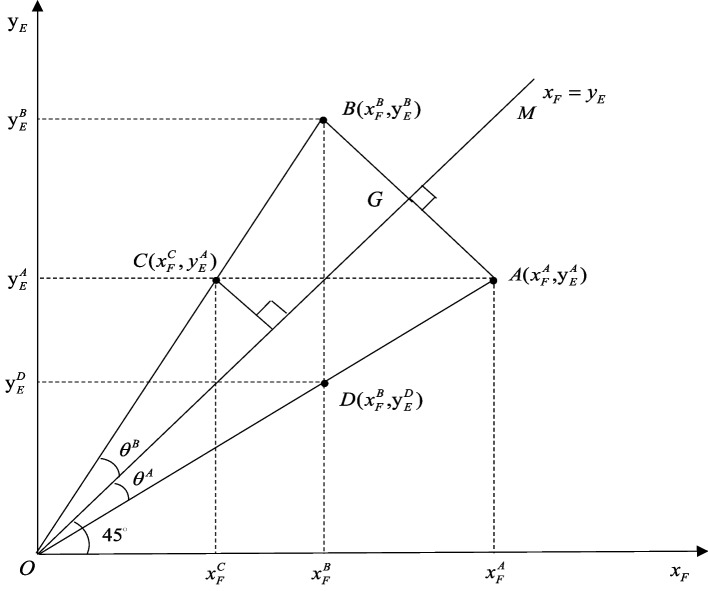
Illustration of the concept of combined efforts in coordination of equity-efficiency development

This study employed an effective scientific method to calculate the combined efforts in the coordination of China’s MHS over time, based on the perspective of common prosperity. China’s MHS is implemented under the policy of common prosperity. Our definition of combined efforts in coordination breaks down the concept of rural–urban equity-efficiency coordination development into two dimensions: “common” and “prosperity.” “Common” means a reduction in the disparities between urban and rural areas in health expenditure burden and health resources allocations [24]. “Prosperity” means achieving higher productivity [25, 26], which is necessary to improve resources allocation through innovations in medical technology and medical institution management and to maximize output efficiency in China’s MHS with a fixed amount of human, financial, and material resources [27, 28]. Maximizing the output efficiency of China’s MHS is the premise and foundation for achieving equitable medical and public health services. Realizing the equity of burden for health expenditure and resources allocation is the goal of continuous improvement in the output efficiency of China’s MHS. To promote high-quality development in the MHS, China must focus more on the equity, efficiency, and mutual promotion relationship of these two factors [29]. Referring to Xu’s [30] concept of assessing deviation in the policy of supply and demand, we incorporated equity and efficiency into the analytical framework and constructed a combined-efforts-in-coordination model to comprehensively evaluate the system operation and explore long-term balanced development, where the horizontal axis represents equity, and the vertical axis represents efficiency (Fig. 1).

In the constructed two-dimensional rectangular coordinate system, the horizontal axis represents the equity of medical and health services ($x_{F}$), and the vertical axis represents the efficiency of the MHS ($y_{E}$). $OM$ is a straight line without deviation and is composed of points where equity and efficiency are equal ($x_{F} { = }y_{E}$), and the angle between the straight line and the horizontal axis is $45^{ \circ }$. $\theta^{A}$ is the angle between $A(x_{F}^{A} ,y_{E}^{A} )$ and the straight line without deviation $OM$, and $\theta^{B}$ is the angle between $B(x_{F}^{B} ,y_{E}^{B} )$ and the straight line without deviation $OM$. The two angles indicate the magnitude of deviation between the actual operating state and the ideal state (coordinated equity-efficiency development) of the current MHS. $AG$ and $BG$ are the distances from $A(x_{F}^{A} ,y_{E}^{A} )$ and $B(x_{F}^{B} ,y_{E}^{B} )$ to the straight line without deviation $OM$ and represent the depth of deviation between the current actual operating state of the MHS and the ideal state. Notably, when $\theta^{A} { = }\theta^{B}$, the efficiency of $A(x_{F}^{A} ,y_{E}^{A} )$ and $C(x_{F}^{C} ,y_{E}^{A} )$ remains the same, but owing to the difference in the extent of equity, there are variations in the actual degree of equity-efficiency coordination and the degree of deviation from the ideal state. Similarly, although the degree of equity of $B(x_{F}^{B} ,y_{E}^{B} )$ and $D(x_{F}^{B} ,y_{E}^{D} )$ is the same, owing to the difference in the level of efficiency, there are variations in the actual degree of equity-efficiency coordination and the degree of deviation from the ideal state.

This study explored the extent and depth of deviation to measure the combined efforts in the coordination of China’s MHS. It used angular deviation to represent the deviation’s breadth and relative distance to represent the deviation’s depth. The combined efforts in coordination can be expressed as

8 $$\lambda_{EF}=\left|\tan\left.(\theta-45^\circ)\right|\right.^\alpha\bullet d_{EF}^\gamma,\theta\in\lbrack0^\circ,90^\circ\rbrack$$

where $\alpha > 0,\gamma > 0$ are the sensitivity coefficients representing the extent and depth of influence on China’s MHS. $d_{EF}$ represents the depth of deviation of the equity-efficiency coordination of the MHS, which is represented by the distance from point $(x_{F} ,y_{E} )$ to the point without deviation (i.e., straight line $x_{F} = y_{E}$), that is, $d_{EF} { = }\frac{{\left| {x_{F} } \right. - \left. {y_{E} } \right|}}{\sqrt 2 }$. We can further simplify the combined efforts in coordination of China’s MHS as follows:

9 $$\lambda_{EF}=2^\frac{-\gamma}2\frac{\left|x_F-\left.y_E\right|^{\alpha+\gamma}\right.}{\left|x_F+\left.y_E\right|^\alpha\right.}$$

The combined-efforts-in-coordination score is between 0 and 1. According to the Pearson correlation coefficient, equity-efficiency coordination is divided into different degrees:$0.0 < \lambda_{EF} < 0.2$ denotes extremely weak migration or no migration; $0.2 < \lambda_{EF} < 0.4$ denotes weak degree migration; $0.4 < \lambda_{EF} < 0.6$ denotes moderate migration; $0.6 < \lambda_{EF} < 0.8$ denotes intensity migration; $0.8 < \lambda_{EF} < 1.0$ denotes extremely strong migration.

### Variables

Six variables were selected in this study to evaluate the equity of China’s MHS. Four variables, including per cash health care expenditure of urban residents, per cash health care expenditure of rural residents, per capita disposable income of urban residents, and per capita disposable income of rural residents, were employed to calculate the health expenditure burden. Two variables, including number of medical professionals per 10,000 people in urban areas and number of medical professionals per 10,000 people in rural areas, were selected to evaluate health resources allocation equity.

In terms of efficiency assessment, three variables, including the number of medical staff, number of medical institutions, and number of medical beds, were selected as inputs. The number of medical staff represents human resources; number of medical institutions represents capital investment; and number of medical beds is an important variable reflecting hardware investment. Another two output variables are the numbers of patients treated and hospitalizations. In addition, three control variables were selected, including the per capita GDP, urbanization rate, and government health expenditure, considering the influence of internal and external factors. All the variables are shown in Table 1.

**Table 1:** Summary statistics of the variables

Category		Variable	Mean	Median	IQR
Equity	burden	Per cash health care expenditure of urban residents (CNY)	691.24	635.71	1754.60
		Per cash health care expenditure of rural residents (CNY)	338.80	186.82	1137.90
		Per capita disposable income of urban residents (CNY)	16,367.42	11,126.25	43,833.80
		Per capita disposable income of rural residents (CNY)	5744.54	3420.95	17,131.50
	allocation	Number of medical professionals per 10,000 people in urban	72.13	64.00	115.00
		Number of medical professionals per 10,000 people in rural	30.20	27.00	52.00
Efficiency	Input	Number of medical staffs (10,000)	803.26	690.27	1347.50
		Number of medical institutions (10,000)	84.00	96.44	107.81
		Number of medical beds (10,000)	466.89	343.96	910.07
	Output	Number of patients treated (100 million)	43.58	25.92	87.20
		Number of hospitalizations (100 million)	1.18	0.75	2.66
	Control	Per capita GDP (CNY)	25,403.77	15,553.00	71,828.00
		Urbanization rate (%)	44.10	43.67	63.89
		Government health expenditure (100 million CNY)	5416.87	1665.70	21,941.90

Data source: Author’s calculations according to National Bureau of Statistics of China

## Results

### Equity of China’s MHS

**Fig. 2:**
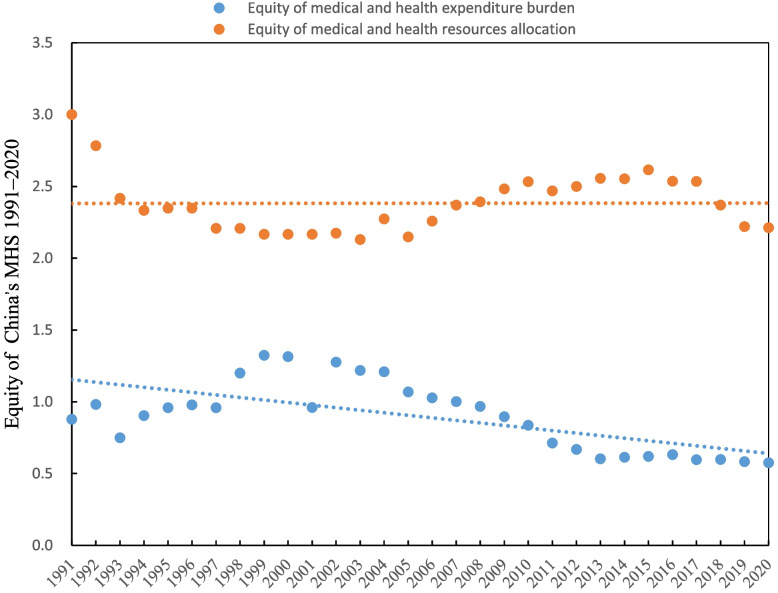
Equity trends of China’s medical and health service system

The equity of China’s MHS in terms of the medical expenditure burden dimension was in a more preferred equity status than that of the health resources allocation dimension. The equity of medical expenditure burden has experienced several fluctuations from 1991 to 2020. From 1991 to 1997, urban residents’ relative responsibility for medical expenditure was much lower than that of rural residents, with $x_{F}^{i}$ less than 1. The equity of health medical expenditure burden significantly improved from 1998 to 2007 because rural residents’ medical expenditure burden was reduced compared with that of urban residents ($x_{F}^{1} > 1$ except for 2000–2001). According to the narrowing trend of $x_{F}^{1}$, the gap in the equity of health medical expenditure burden slowly widened after 2007. The medical and health resources allocation indicates an alarming inequity in urban and rural China during the entire period, with the number of medical professionals per 10,000 people in urban areas being 2.130–2.783 times as many as the number of medical professionals per 10,000 people in rural areas. The equity results are shown in Table 2, and the equity trend is shown in Fig. 2.

**Table 2:** Equity, efficiency and combined efforts in coordination of China’s MHS 1991–2020

Year	$x_{F}^{1}$	$x_{F}^{2}$	$y_{E}$	$\lambda_{EF}^{1}$	$\lambda_{EF}^{2}$
1991	0.878	3.000	1.000	0.045	0.744
1992	0.982	2.783	0.996	0.005	0.665
1993	0.749	2.417	0.953	0.076	0.544
1994	0.904	2.333	0.978	0.027	0.504
1995	0.959	2.348	0.989	0.011	0.505
1996	0.979	2.348	0.581	0.148	0.657
1997	0.959	2.208	0.567	0.146	0.610
1998	1.200	2.208	0.544	0.244	0.619
1999	1.324	2.167	0.531	0.295	0.608
2000	1.314	2.167	0.546	0.286	0.603
2001	0.961	2.167	0.547	0.154	0.603
2002	1.277	2.174	0.613	0.247	0.581
2003	1.218	2.130	0.617	0.224	0.563
2004	1.209	2.273	0.654	0.206	0.602
2005	1.068	2.148	0.684	0.143	0.544
2006	1.027	2.259	0.723	0.113	0.571
2007	1.001	2.370	0.851	0.056	0.565
2008	0.968	2.393	0.915	0.020	0.550
2009	0.896	2.483	1.000	0.039	0.552
2010	0.837	2.533	0.990	0.057	0.574
2011	0.713	2.469	0.995	0.105	0.548
2012	0.669	2.500	1.000	0.123	0.558
2013	0.603	2.556	1.000	0.148	0.579
2014	0.614	2.553	1.000	0.144	0.578
2015	0.620	2.615	0.989	0.137	0.605
2016	0.632	2.537	0.997	0.136	0.573
2017	0.597	2.535	1.000	0.150	0.571
2018	0.599	2.370	1.000	0.149	0.510
2019	0.583	2.220	1.000	0.155	0.454
2020	0.576	2.212	0.865	0.107	0.501
Mean	0.897	2.383	0.838	0.130	0.575

Data source: Author’s calculations according to National Bureau of Statistics of China

### Efficiency of China’s MHS

Table 3 presents the efficiency of China’s MHS from 1991 to 2020. The average scores of overall efficiency, technical efficiency and scale efficiency were 0.838, 0.973, and 0.859, respectively. From 1991 to 2020, eight years (26.67%), including 1991, 2009, 2012–2014, and 2017–2019, had an overall efficiency score of 1, indicating that China’s MHS in these years was relatively efficient. Eight years (26.67%), including 1992–1995, 2007, 2008, 2011, and 2016, had a technical efficiency score of 1 but an overall or scale efficiency score of less than 1, indicating that the efficiency of China’s MHS in these years was weak when compared with efficient years. Additionally, the other 14 years (46.67%) had overall efficiency, technical efficiency, and scale efficiency scores of less than 1, suggesting that China’s MHS in these years was inefficient. China’s MHS was seriously inefficient from 1996 to 2006.

**Table 3:** Efficiency of China’s MHS from 1991 to 2020

Year	Overall efficiency	Technical efficiency	Scale efficiency	Type of scale inefficiency	Relatively efficiency status
1991	1.000	1.000	1.000	-	Efficient
1992	0.996	1.000	0.995	irs	Weakly efficient
1993	0.953	1.000	0.999	-	Weakly efficient
1994	0.978	1.000	1.000	-	Weakly efficient
1995	0.989	1.000	0.997	irs	Weakly efficient
1996	0.581	0.925	0.628	irs	Inefficient
1997	0.567	0.926	0.609	irs	Inefficient
1998	0.544	0.912	0.596	irs	Inefficient
1999	0.531	0.908	0.587	irs	Inefficient
2000	0.546	0.934	0.595	irs	Inefficient
2001	0.547	0.916	0.597	irs	Inefficient
2002	0.613	0.965	0.635	irs	Inefficient
2003	0.617	0.963	0.641	irs	Inefficient
2004	0.654	0.962	0.680	irs	Inefficient
2005	0.684	0.968	0.702	irs	Inefficient
2006	0.723	0.958	0.755	irs	Inefficient
2007	0.851	1.000	0.839	irs	Weakly efficient
2008	0.915	1.000	0.912	irs	Weakly efficient
2009	1.000	1.000	1.000	irs	Weakly efficient
2010	0.990	0.997	0.999	irs	Inefficient
2011	0.995	1.000	0.999	irs	Weakly efficient
2012	1.000	1.000	1.000	-	Efficient
2013	1.000	1.000	1.000	drs	Efficient
2014	1.000	0.979	1.000	drs	Weakly efficient
2015	0.989	0.998	0.999	-	Inefficient
2016	0.997	1.000	1.000	-	Weakly efficient
2017	1.000	1.000	1.000	-	Efficient
2018	1.000	1.000	1.000	-	Efficient
2019	1.000	1.000	1.000	-	Efficient
2020	0.865	0.865	0.997	-	Inefficient
Mean	0.838	0.973	0.859	/	/

Data source: Author’s calculations according to National Bureau of Statistics of China

### Combined efforts in coordination of China’s MHS

Table 2 presents the combined efforts in the coordination of China’s MHS from 1991 to 2020. The average scores of the combined efforts in coordination in terms of medical expenditure burden dimension and combined efforts in coordination in terms of the health resources allocation dimension were 0.074 and 0.375, respectively. In terms of the medical expenditure burden dimension, 24 years (80.00%) had a score between 0 to 0.2, indicating that equity-efficiency coordination was extremely weak in these years, migration or no migration. Additionally, the other six years had a score between 0.2 to 0.4, indicating that the equity-efficiency coordination was weak due to migration in these years. In terms of medical and health resources allocation dimensions, 20 years (66.67%) had a score between 0.4 to 0.6, indicating that the equity-efficiency coordination was moderate due to migration in these years. The other 10 years (33.33%) had a score between 0.6 to 0.8, indicating that the equity-efficiency coordination was extremely strong due to migration in these years.

## Discussion

The evaluation of China’s MHS’s equity showed that the equity values by medical expenditure burden were all better than those by medical and health resources allocation, suggesting that a larger disparity exists in the medical and health resources allocation of China’s MHS than in the medical expenditure burden. One possible explanation for this finding is that the equity in medical expenditure burden between urban and rural areas relates closely to health-system reforms in China. From 1991 to 1997, rural residents’ medical expenditure burden was much higher than that of urban residents. The reason was that rural residents had almost no access to medical insurance. From 1998 to 2002, China promoted employee medical insurance; however, unemployed urban and rural residents still did not have a robust health care system. Nonetheless, the gap in equity between urban and rural areas slowed down during these years. The possible cause for this finding is that treatment prices and health care costs rapidly increased in urban areas. In 2003, rural residents’ medical expenses were significantly reduced after the implementation of the New Rural Cooperative Medical System.

Given that a proportion of rural residents may need to travel to the city to access necessary examinations and treatment due to the inequity of China’s MHS by health resources allocation, the improvement of their health outcomes is affected [31]. Policymakers must consider the number of medical professionals in different regions when planning medical service resources allocation in urban and rural regions [32]. Medical professionals are the backbone of China’s MHS, playing a vital role in providing health services for urban and rural residents. However, the number of medical professionals in underdeveloped areas are insufficient per capita, which is also the case globally [33, 34]. Increasing the number of well-educated and trained medical professionals will be key to achieving equity in the health resources allocation dimension.

More than one-third of the years had overall efficiency, technical efficiency, and scale efficiency scores of less than 1. Inefficiency and regional differences in China’s MHS have also been found in many previous studies. The efficiency of government medical and health expenditure in China has obvious regional differences. The more misallocated healthcare resources are, the lower is the efficiency [35, 36]. The efficiency of primary healthcare services has significant differences among the 31 provinces [37]. The efficiency of China’s MHS was deteriorating before 1999, the rural cooperative medical system dropped to the lowest level, labor health insurance and government health insurance were failing, medical insurance coverage was shrinking, and scale efficiency levels continued to show negative growth trajectories. Technical progress and efficiency, as well as allocation efficiency, were influenced by China’s comprehensive healthcare system reform during the planned economy period. After 1999, China’s MHS became more efficient, which increased by an average of 2.51% per year. The resources allocation capacity of China’s MHS was enhanced, and the utilization of various input elements improved. The implementation of the new rural insurance system in 2009, the realization of universal medical insurance in 2010, and the implementation of critical illness insurance for urban and rural residents in 2012 allowed China’s MHS to develop gradually and efficiently. It remained stable at a high level except for 2020, which was anomalous.

### Combined efforts in coordination

An in-depth analysis of the combined efforts in coordination may deepen our understanding of the internal logic of the MHS’ overall operations. It is important to note that when the equity-efficiency index is equal, equity and efficiency can have different combinations. For example, equity-efficiency indexes for 1996 and 2013 are equal (0.148) in medical expenditure burden dimensions, indicating that the equity-efficiency coordination degree of China’s MHS during both periods was also equal. However, equity and efficiency levels in 2013 were both higher than those in 1996. The combined efforts in coordination in 1993 and 2005 are also the same (0.544) in the health resources allocation dimension. Equity in 2005 was higher than that in 1993, while efficiency in 2005 was lower than that in 1993. The main reason is the difference in the political environment for the coordinated development of equity efficiency in urban and rural China’s MHS. The trade-off between equity and efficiency was a political decision rather than a technical concept. From 1998 to 2007, the focus of China’s MHS construction was to realize the equity of medical burden between urban and rural areas, and a series of policies were implemented. These included the Urban Employee Basic Medical Insurance Scheme, which was promoted nationwide in 1998; the New Rural Cooperative Medical System, which was established in 2003; and the Urban Resident Basic Medical Insurance Scheme, which expanded its coverage to the urban unemployed in 2007. Equity was significantly improved by efficiency loss, especially scale efficiency loss at that stage.

### Limitations

This study explored the equity, efficiency, and equity-efficiency coordination of health resources allocation and explored the problems causing inequity, inefficiency, and uncoordination. A more targeted policy can be developed via analysis of the problems in China’s MHS. This study has some limitations. One limitation was the difficulties in obtaining health indicators stratified according to urban and rural locations in China, making it harder to include more variables in the efficiency model. Another limitation was the scope of the study, limited to hospital information. Perhaps if we were able to use Primary Health Care data, we would have obtained very different results.

## Conclusion

The equity of China’s MHS medical expenditure burden dimension is superior in the health resources allocation dimension, and scale insufficiency is the main cause of inadequate overall efficiency. This study provides valuable information for policymakers. The government should provide medical subsidies for rural residents to adjust medical expenditure burden levels in both urban and rural areas, control the differences in medical expenditure burden between urban and rural residents to a reasonable range, and continuously improve the equity level of urban and rural residents in the medical expenditure burden dimension. The government should also raise salaries for medical professionals in rural areas, improve the working environment for rural medical institutions, and guide the rational flow of medical professionals between urban and rural areas. Improving the efficiency of China’s MHS requires technical innovation and an improvement in management levels in the medical industry. Central and local governments can encourage innovation by increasing investment in medical technology innovation. Regarding management, China can strengthen the supervision of investment funds and replace traditional bureaucratic enforcement strategies with flexible management strategies.

## Data Availability

The datasets used and analyzed during the current study are available from the corresponding author on reasonable request.

## Abbreviations

BCC: Banker-Charnes-Cooper model
DEA: Data envelopment analysis
EFFCH: Efficiency change
MPI: Malmquist productivity index
MHS: Medical and Health Service System
PECH: Pure efficiency change
SECH: Scale efficiency change
TECHCH: Technical change
